# A Glyoxalase-1 Knockdown Does Not Have Major Short Term Effects on Energy Expenditure and Atherosclerosis in Mice

**DOI:** 10.1155/2016/2981639

**Published:** 2015-12-15

**Authors:** Markus Wortmann, Maani Hakimi, Thomas Fleming, Andreas S. Peters, Tjeerd P. Sijmonsma, Stephan Herzig, Peter P. Nawroth, Dittmar Böckler, Susanne Dihlmann

**Affiliations:** ^1^Department of Vascular and Endovascular Surgery, University of Heidelberg, 69120 Heidelberg, Germany; ^2^Department of Medicine I and Clinical Chemistry, University of Heidelberg, 69120 Heidelberg, Germany; ^3^Joint Research Division, Molecular Metabolic Control, German Cancer Research Center DKFZ, Network Aging Research, ZMBH, and University Hospital Heidelberg, 69120 Heidelberg, Germany

## Abstract

*Objective*. Glyoxalase-1 is an enzyme detoxifying methylglyoxal (MG). MG is a potent precursor of advanced glycation endproducts which are regarded to be a key player in micro- and macrovascular damage. Yet, the role of Glo1 in atherosclerosis remains unclear. In this study, the effect of Glo1 on mouse metabolism and atherosclerosis is evaluated.* Methods*. Glo1 knockdown mice were fed a high fat or a standard diet for 10 weeks. Body weight and composition were investigated by Echo MRI. The PhenoMaster system was used to measure the energy expenditure. To evaluate the impact of Glo1 on atherosclerosis, Glo1^KD^ mice were crossed with ApoE-knockout mice and fed a high fat diet for 14 weeks.* Results*. Glo1 activity was significantly reduced in heart, liver, and kidney lysates derived from Glo1^KD^ mice. Yet, there was no increase in methylglyoxal-derived AGEs in all organs analyzed. The Glo1 knockdown did not affect body weight or body composition. Metabolic studies via indirect calorimetry did not show significant effects on energy expenditure. Glo1^KD^ mice crossed to ApoE^−/−^ mice did not show enhanced formation of atherosclerosis.* Conclusion*. A Glo1 knockdown does not have major short term effects on the energy expenditure or the formation of atherosclerotic plaques.

## 1. Introduction

Diabetes is one of the major risk factors in the development of atherosclerosis. However, the link between diabetes and atherosclerosis is still poorly understood. Increasing evidence suggests that dicarbonyl stress, the abnormal accumulation of *α*-oxoaldehyde metabolites during hyperglycemic metabolism, contributes to an increased risk of cardiovascular disease [1]. A recent study with more than 90.000 patients and controls identified the glyoxalase-1 (Glo1) gene as a key driver of coronary artery disease [2].

Glo1 is a cytosolic protein that forms, together with glyoxalase 2 and glutathione, the glyoxalase system. The main function of this system is the detoxification of reactive dicarbonyls, in particular methylglyoxal (MG), which forms from spontaneous degradation of triosephosphates [3]. MG modifies arginine residues of proteins, resulting in formation of so-called advanced glycation end products (AGEs). An age-dependent decline in Glo1 activity has been shown to cause increased MG plasma levels and formation of AGEs [3]. Moreover, both, accumulation of MG and increased AGE levels, have been shown to be involved in a number of several microvascular complications of diabetes mellitus, for example, nephropathy [4] and retinopathy [5].

As recently reviewed by us [6], there is increasing evidence that the balance between production of MG and its detoxification by Glo1 might be involved in the formation and progression of atherosclerotic lesions. In addition, chemical inhibition of Glo1 has been shown to induce atherosclerosis in ApoE deficient mice [7]. In this study, Glo1 was further investigated in an* in vivo* model of Glo1 knockdown (Glo1^KD^) mice, to determine its distinct role in metabolism under different mouse diets and progression of atherosclerosis.

## 2. Materials and Methods

### 2.1. Animals and Genotyping

The transgenic mouse line B6.129P2-*Apoe* ^*tm1Unc*^ (ApoE^−/−^) was purchased from Jackson laboratories; the transgenic Glo1 knockdown mice (Glo1^KD^) were a kind gift of the lab of Michael Brownlee [8]. For the atherosclerosis experiments, Glo1^KD^ mice were bred onto the ApoE^−/−^ mice. Male double transgenic mice (Glo1^KD^ × ApoE^−/−^) were compared to age-matched male ApoE^−/−^ and Glo1^KD^ littermates. For genotyping, genomic DNA was extracted from mouse tails and analyzed by PCR. Primers used are listed in Supplementary Table 1 (see Supplementary Table 1 in the Supplementary Material available online at http://dx.doi.org/10.1155/2016/2981639). Animals were fed a standard chow for 10 weeks. Subsequently, half the animals in each group (*n* = 12) were placed on a high fat diet for another 14 weeks to induce obesity and atherosclerosis as indicated in the figures and text. Animals receiving standard chow diet (*n* = 12) for the entire experimental period were used as controls.

### 2.2. Analysis of Feeding Behavior, Locomotor Activity, and Metabolic Parameters

Ten-week-old wild-type and Glo1^KD^ mice were fed experimental diets with 60% calories derived from fat (in the following referred to as high fat diet) and 10% calories derived from fat (in the following referred to as low fat diet) (resp., D12492 or D12450B, Research Diets, New Brunswick) for 10 weeks (= four groups with five animals each). Body weight was monitored weekly for eight weeks until the mice entered the PhenoMaster System (TSE systems GmbH, Bad Homburg, Germany). After leaving the PhenoMaster System, an Echo-MRI analysis (Echo Medical System, Houston, TX, USA) was applied to determine the body composition of the mice. Data analysis was performed as described [9].

### 2.3. Tissue Preparation and Quantification of Glo1 Activity

Heart, liver, and kidneys were removed from 22-week-old Glo1^KD^ mice and wild-type littermates, both fed with a standard chow diet. Tissues were immediately flash frozen in liquid nitrogen and stored at −80°C. Tissue homogenization, preparation of total protein extracts, and analysis of Glo-1 activity were performed as previously described [10].

### 2.4. Glo1 Western Blotting

Protein extracts (20 *μ*g) were separated under denaturing conditions on precast 4% to 20% mini gels, transferred to a nitrocellulose membrane, and blocked with 5% non-fat dry milk. Membranes were incubated overnight with rabbit-anti-Glo1 (1 : 1000; ab96032, Abcam, Cambridge, UK) or for one hour with mouse-anti-actin (1 : 5000; MP Biomedicals, Eschwege, Germany). Membranes were subsequently incubated with the appropriate horseradish peroxidase conjugated secondary antibody (1 : 5000; Jackson ImmunoResearch Laboratories, Europe; Dianova, Hamburg, Germany). Immunoreactive proteins were visualized on X-ray films using enhanced chemiluminescence detection reagents, according to the manufacturer's instructions. Densitometric analysis (ImageJ) was used to determine relative Glo1 levels, normalized to actin as a loading control.

### 2.5. Analysis of Methylglyoxal Derivatives

Frozen excised tissues from heart, liver, and kidneys were ground to a fine powder under liquid nitrogen and resuspended in ice cold lysis buffer (60 mM Na2HPO4, 40 mM NaH2PO4, 10 mM KCl, 1 mM MgSO4, pH 7.0). Extracts were incubated on ice for 10 min, homogenized with a syringe and a fine needle, and centrifuged (18000 g, 4°C). Supernatants were used for analysis of methylglyoxal-derived AGEs by ELISA (OxiSelect methylglyoxal (MG) competitive ELISA Kit, Cell Biolabs Inc., San Diego, USA) according to the recommendations of the manufacturer. Briefly, undiluted cell lysates were loaded to MG-conjugate coated plates and detected by Anti-MG antibody, secondary antibody, and substrate solution. Absorbance was read in a microplate reader at 450 nm as primary wave length.

### 2.6. Blood Glucose and Plasma Lipid Quantification

Blood was sampled from the heart in EDTA-coated microvettes to prevent coagulation. Blood glucose was determined immediately with fresh blood by using an Accu Check Aviva blood glucose meter (Roche, Mannheim, Germany). After centrifugation, plasma was removed and stored at −80°C until further analysis. Triglycerides and cholesterol were determined as previously described [11] using standard analysis methods at the Central Diagnostics Laboratory of the University Hospital Heidelberg.

### 2.7. Characterization and Quantification of Atherosclerotic Lesions

En face preparations of the aortic arch were performed following a standard protocol. Briefly, mice were lethally anesthetized using inhalation anesthesia (isoflurane) and the complete aortas were removed together with the heart. Using a stereo microscope, aortas were dissected from any perivascular fat and connective tissue and subsequently sliced longitudinally. Aortas were fixed in 80% 2-propanol and stained with oil red O (Sigma-Aldrich, Steinheim, Germany; 0.5% in 2-propanol) for 10 min. After differentiating in 80% 2-propanol for 2–5 min, en face pictures were taken for digital analysis. ImageJ was used to determine red stained areas in relation to the total area of the aortic arch or the complete aorta, respectively. For analysis of the aortic sinus, the upper part of the heart was formalin-fixed and embedded in paraffin. Transversal sections of the aortic sinus were prepared and MOVAT staining was performed (detailed staining protocols are available on request). Quantification of atherosclerosis was performed by analysis of MOVAT-stained cross sections of the aortic sinus. ImageJ was used to determine the remaining lumen in relation to the total area of the aortic sinus.

### 2.8. Immunohistochemical Detection of Glo1

Serial 2 *μ*m transversal sections were prepared from formalin-fixed and paraffin-embedded aortic sinus specimens. After deparaffinization and rehydration, sections were pretreated in target retrieval solution pH 6.0 (DAKO, Glostrup, Denmark) in a steamer for 30 min. After 5 min washing in TBS buffer (20 mM Tris, 137 mM NaCl, pH 7.6) sections were incubated overnight with anti-Glo1 (1 : 300; ab96032, Abcam, Cambridge, UK). The DAKO real detection system peroxidase/AEC Rabbit/Mouse (DAKO, Glostrup, Denmark), containing biotinylated secondary antibody, streptavidin-HRP, and AEC/H2O2 substrate, was applied for further treatment, following the manufacturer's recommendation. Counterstaining was performed for 30 sec with Mayer's hemalum solution (Merck Darmstadt, Germany).

### 2.9. Statistical Analysis

Statistical analysis was performed using SigmaStat 2.0 software (Jandel GmbH, Erkrath, Germany) or SPSS Statistics version 22 (IBM, Armonk, USA). Experimental values are expressed as mean ± SD unless otherwise stated. Statistically significant differences between groups were calculated by Student's *t*-test or two-way ANOVA. Values of *p* < 0.05 were considered significant.

## 3. Results

### 3.1. The Glo1 Knockdown Results in a Significantly Reduced Protein Level in the Kidney and a Significantly Reduced Glo1 Activity in Heart, Kidney, and Liver

**Figure 1:**
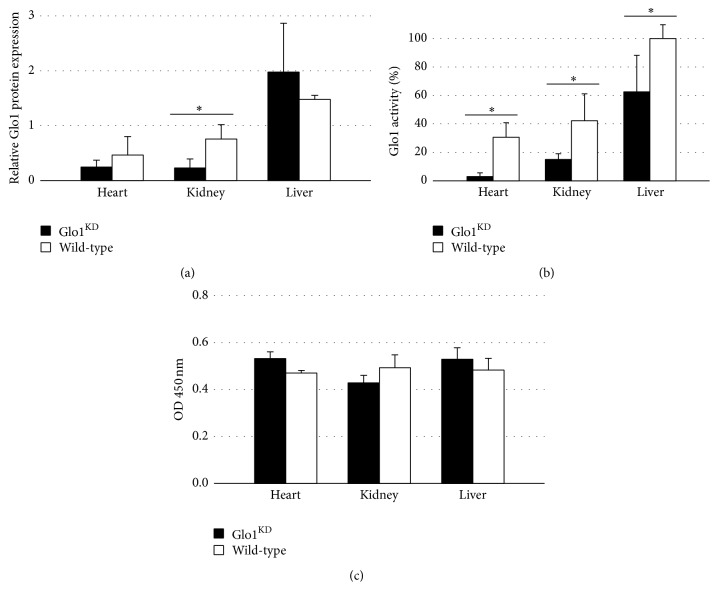
(a) Glo1 protein levels measured via Western blot were significantly reduced in the kidney (*p* = 0.0096), whereas protein levels in the heart (*p* = 0.27) and the liver (*p* = 0.34) did not differ significantly. (b) Glo1 activity was significantly reduced in the heart (*p* = 0.001), kidney (*p* = 0.03), and liver (*p* = 0.03) of Glo1^KD^ mice in comparison to wild-type animals (*n* = 4). Glo1 activity in the liver of wild-type mice was arbitrarily set to 100%. (c) The levels of methylglyoxal-derived AGEs were measured via an ELISA. There was no significant impact of the Glo1 knockdown on the methylglyoxal-derived AGEs in extracts of heart, kidney, and liver (*p* ≥ 0.05).

Adult Glo1^KD^ mice were sacrificed and Glo1 protein levels as well as Glo1 activity were measured in the heart, liver, and kidney. Glo1 protein levels were measured via Western blotting in relation to actin. Glo1 protein levels were significantly reduced in kidney (Glo1^KD^ versus wild-type: 0.231 ± 0.165 versus 0.757 ± 0.264, *p* = 0.0096), whereas protein levels in heart (Glo1^KD^ versus wild-type: 0.246 ± 0.128 versus 0.467 ± 0.335, *p* = 0.27) and liver (Glo1^KD^ versus wild-type: 1.977 ± 0.892 versus 1.480 ± 0.072, *p* = 0.34) were not significantly different (Figure 1(a)).

Glo1 activity was reduced in liver lysates by 38% (62.5% ± 25.7% versus 100% ± 9.7%,*p* = 0.03), in kidney lysates by 64% (15.1 ± 3.9% versus 42.2 ± 18.8%, *p* = 0.03), and in heart lysates by 90% (3 ± 2.6% versus 30.6 ± 10.1%, *p* = 0.001) in comparison to wild-type mice. Glo1 activity in the liver of wild-type mice was arbitrarily set to 100% (Figure 1(b)).

### 3.2. Levels of Methylglyoxal-Derived AGEs Were Not Elevated in Glo1^KD^ Mice

In order to measure the levels of methylglyoxal-derived AGEs, an ELISA measuring methylglyoxal-hydroimidazolone was performed. Although Glo1 activity was significantly reduced in all organs analyzed, the levels of methylglyoxal-derived AGEs were not affected in liver (high fat diet: 2.8 ± 0.36 *μ*g/mL versus 3.3 ± 0.42 *μ*g/mL, *p* = 0.077; standard chow: 2.1 ± 0.33 *μ*g/mL versus 3.1 ± 0.09 *μ*g/mL, *p* = 0.13), kidney (high fat diet: 2.9 ± 0.33 *μ*g/mL versus 2.2 ± 0.3 *μ*g/mL, *p* = 0.061; standard chow: 2.94 ± 0.29 *μ*g/mL versus 4.3 ± 0.65 *μ*g/mL, *p* = 0.076), and heart (high fat diet: 5.5 ± 0.91 *μ*g/mL versus 7.5 ± 1.1 *μ*g/mL, *p* = 0.36; standard chow: 5.8 ± 0.97 *μ*g/mL versus 8.0 ± 1.25 *μ*g/mL, *p* = 0.091) lysates (Figure 1(c)).

### 3.3. Glo1^KD^ Mice Do Not Show Major Alterations in Feeding Behavior, Locomotor Activity, and Different Metabolic Parameters

**Figure 2:**
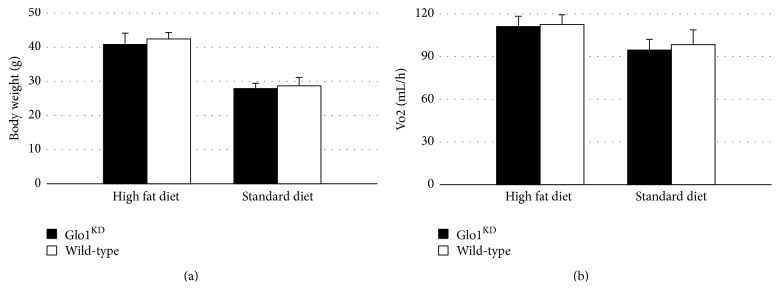
(a) The mean body weight did not differ between Glo1^KD^ mice and wild-type mice, neither on high fat diet (*p* = 0.654) nor on standard diet. (b) The mean oxygen consumption over 24 h was measured using the PhenoMaster system. The genotype did not have a significant influence (*p* ≥ 0.05).

Glo1^KD^ mice as well as C57Bl/6 mice, which served as control animals, were fed a standard chow or alternatively a high fat diet for 10 weeks (5 mice per groups). During this time the body weight was measured weekly. The weight gain of the Glo1^KD^ mice, in relation to the weight at the beginning of the experiment, did not differ from the weight gain of wild-type mice after 10 weeks (Glo1^KD^ versus wild-type, standard chow: 154 ± 7% versus 143 ± 5%, *p* = 0.241; Glo1^KD^ versus wild-type, high fat diet: 213 ± 11% versus 220 ± 10%, *p* = 0.654). The same was true for the absolute body weight after 10 weeks (Glo1^KD^ versus wild-type, standard chow: 40.8 ± 3.32 g versus 42.4 ± 1.96 g, *p* = 0.379; Glo1^KD^ versus wild-type, high fat diet: 27.9 ± 1.57 g versus 28.7 ± 2.44 g, *p* = 0.547) (Figure 2(a)).

Ten weeks after the beginning of the experiment, feeding behavior, locomotor activity, and the energy expenditure were measured using the PhenoMaster system. The knockdown of Glo1 did not cause any major metabolic alterations, regarding neither oxygen consumption (Figure 2(b)) nor food intake or activity (Supplemental Figure S1).

After eleven weeks, nuclear magnetic resonance (NMR) relaxometry was performed via the Echo-MRI system to analyze the body composition. There was no significant difference in the total bodyweight or in the relative fat or lean weight (*p* ≥ 0.05) (Table 1).

### 3.4. Double Transgenic Glo1^KD^ × ApoE^−/−^ Mice Do Not Suffer from Enhanced Atherosclerosis

**Figure 3:**
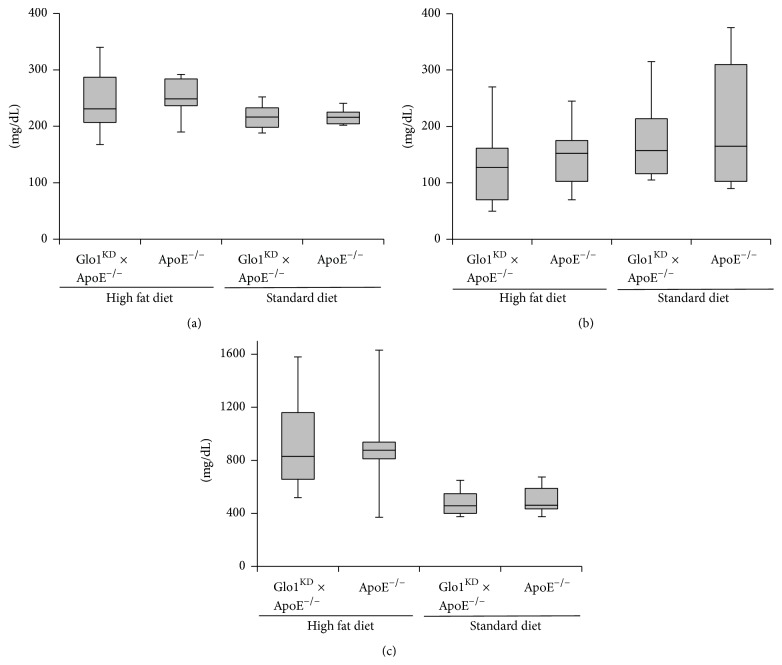
Blood glucose (a), triglycerides (b), and cholesterol (c) were measured in Glo1^KD^ × ApoE^−/−^ and ApoE^−/−^ mice, both on a high fat and on a standard diet. The Glo1 activity did not have an impact on all parameters measured.

**Figure 4:**
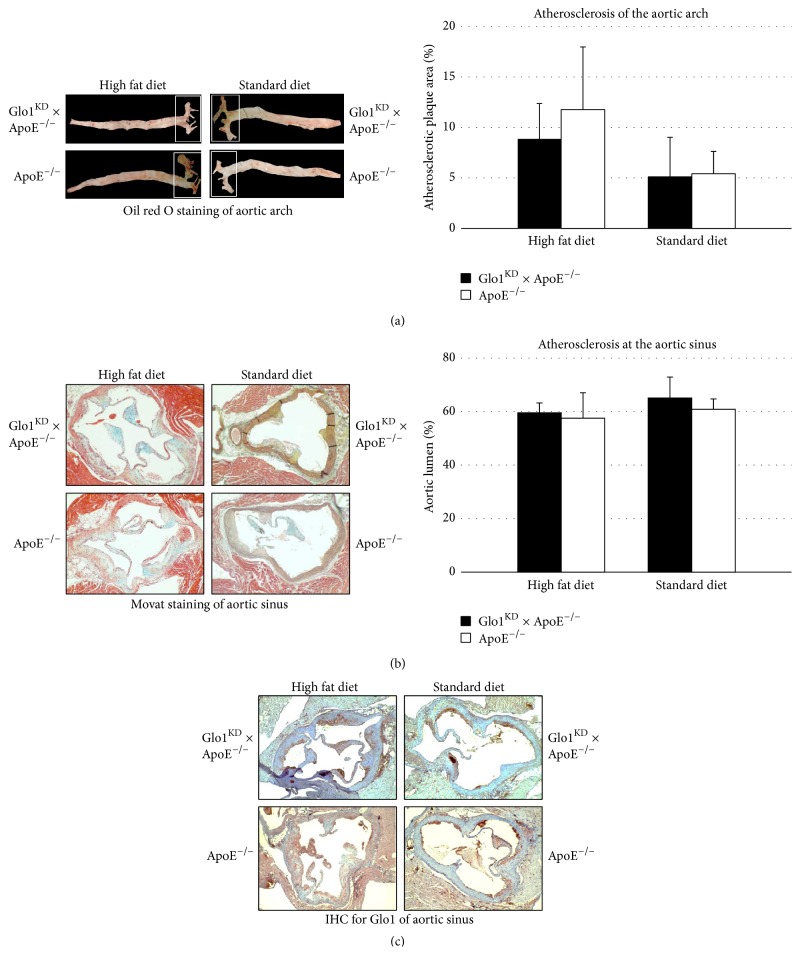
(a) En face preparations were performed in order to quantify the amount of atherosclerotic plaques. Bars refer to the mean of oil red O positive areas, analyzed in relation to the total area of the aortic arch ± standard deviation. The knockdown of Glo1 did not show any significant effect on the formation of atherosclerotic plaques (*p* ≥ 0.05). (b) Transversal sections of the aortic sinus were stained via a MOVAT staining in order to analyze the atherosclerotic plaque formation. Bars represent the mean of the aortic lumina (as determined by ImageJ analysis, lumen area in relation to the total area surrounded by the outer media). The Glo1 knockdown did not have a significant impact of the stenosis caused by atherosclerotic plaques (*p* ≥ 0.05). (c) Immunohistochemical detection of Glo1 performed with transversal sections of the aortic sinus. Glo1 was mainly detected in the atherosclerotic plaques.

The double transgenic (Glo1^KD^ × ApoE^−/−^) mice received a high fat diet for 10 weeks in order to induce formation of atherosclerotic plaques. The Glo1 knockdown did not cause alterations in the triglyceride, cholesterol, or blood sugar levels (Figures 3(a), 3(b), and 3(c)). Also the gain of weight was not affected by the Glo1 knockdown (*data not shown*). With respect to atherosclerosis, the knockdown of Glo1 did not enhance the formation of atherosclerotic plaques in the aortic arch analyzed via en face preparations (Figure 4(a)). Similar findings were also obtained for en face preparations of the whole aorta (Supplemental Figure 2) and the plaque area in histological sections of the aortic sinus (Figure 4(b)). Immunohistochemical analysis of the aortic sinus for detection of Glo1 expression revealed a similar distribution of Glo1-positive areas in all phenotypes, irrespective of the diet. Glo1 was mainly detected within the plaques and the myocardium, whereas little or no Glo1 expression was detected within the aortic wall (Figure 4(c)).

## 4. Discussion

A reduced capacity of Glo1 to detoxify methylglyoxal is associated with endothelial dysfunction, nephropathy, and neuropathy, all of which represent important features of microvascular complications associated with diabetes mellitus.

However, the function of Glo1 in macrovascular disease, particularly atherosclerosis, still remains uncertain [6]. While large epidemiologic studies have not been able to detect effects of different SNPs of the Glo1 gene on formation of atherosclerosis [12, 13], Hanssen et al. reported higher levels of advanced glycation end products in human carotid atherosclerotic plaques associated with a higher risk of plaque rupture [14].

Even less is known about the role of Glo1 in metabolism, particularly in hyperlipidaemia being one of the main risk factors for atherosclerosis.

The aim of this study was to specify the role of Glo1 in mouse metabolism under the challenge of different diets (high fat diet versus standard chow) and its role in the formation of atherosclerotic plaques. Considering its role in detoxifying methylglyoxal, a potent precursor of MG-derived AGEs, we expected an enhanced formation of atherosclerotic plaques in Glo1^KD^ mice. Furthermore, significant changes to the mouse metabolism were assumed.

In this study, the Glo1 knockdown caused a significant reduction of Glo1 protein levels in tissue extracts derived from the kidney, whereas protein levels in heart and liver extracts were not affected. Nevertheless, Glo1 activity was significantly diminished in all organ extracts named by 38%–90% which agrees well with previously published data for the same strain of Glo1^KD^ mice [8].

Since the Glo1 activity assays have been performed under* in vitro* conditions, supplemented with reduced glutathione, we cannot conclude from our data whether the reduced Glo1 activities are reflected under* in vivo* conditions as well, and whether they resulted in increased accumulation of AGEs.

The levels of MG-derived AGEs were determined via a MG-H1 ELISA in the lysates of kidney, liver, and heart. Surprisingly, MG-derived AGE levels were unaffected in the knockdown mice. This was in line with data from Geoffrion et al., who did not detect increased AGE levels, represented by glycated aortal collagen, in the same mouse strain [15]. It should be noted however, that this study is slightly limited by the use of imprecise methods for determination of MG-derived AGEs since the ELISA technique used is hardly reproducible and not quantitative. Future analysis, using analytical mass spectrometry, will help to better define and quantify the accumulation of MG-derived AGEs.

Glo1^KD^ mice did not show any major metabolic aberrations. Neither in regard to surrogate parameters, such as oxygen consumption or resting metabolic rate (analyzed in metabolic cages), nor in the body weight or body composition, which were measured by Echo-MRI, a significant impact of the Glo1 knockdown was detectable. Even challenging the metabolism with a high fat diet, bearing in mind that more methylglyoxal may be accumulating during hyperlipidaemic metabolism, did not reveal any impact of Glo1 on mouse metabolism.

In order to study the role of Glo1 in the progress of atherosclerosis, double transgenic Glo1^KD^ × ApoE^−/−^ mice were created. These mice, compared to age-matched ApoE^−/−^ mice, did not show any significant difference in weight, cholesterol, or triglyceride levels after being fed a high fat diet or standard chow for 10 weeks.

The ApoE^−/−^ mouse strain used in this experiment mainly suffers from atherosclerotic lesions in the aortic sinus as well as in the proximal aorta [16, 17]. Increased AGE accumulation caused by reduction of Glo1 activity should thus result in larger or faster plaque formation. In contrast, the Glo1 knockdown in double transgenic mice did not have any impact on atherosclerosis of the upper aorta. Atherosclerotic lesions in the infrarenal aorta were scarcely observed in all mouse strains, and additional analysis of atherosclerotic plaques of the complete aorta did not show an impact of the Glo1 knockdown, either.

While in this study the Glo1 activity was reduced via a knockdown model, Hanssen et al. have crossed Glo1 overexpressing mice with ApoE^−/−^ mice in order to evaluate whether a higher Glo1 activity might reduce the formation of atherosclerotic plaques. Consistent with our results, there was no protective effect of the Glo1 overexpression, neither under regular conditions nor under diabetic conditions [18].

During the preparation of this paper, Geoffrion et al. have published a study in which both Glo1 knockdown mice and Glo1 overexpressing mice were evaluated with regard to the formation of atherosclerotic lesions [15]. In line with our results, neither an enhanced nor a reduced Glo1 activity did have any effects on atherosclerosis, even after streptozotocin induced diabetes.

Considering the lack of MG-derived AGE accumulation in the Glo1^KD^ mice of our study, the reduction in Glo1 activity may not suffice to cause pathological consequences in macrovascular disease. Alternatively, the reduced detoxification rate by Glo1 might be replaced by other enzymatic reactions. For example, aldo-keto reductase has recently been shown to significantly contribute to MG-detoxification in various cell types, particularly when glutathione is limited in tissues and oxidative stress is increased [19, 20]. It will be interesting to see whether this enzyme or other mechanisms can overcome effects of reduced Glo1 activity in the context of atherosclerosis. Future studies using Glo1 knockout mice with a complete reduction of the Glo1 activity will be needed in order to finally work out the role of Glo1 in atherosclerosis and metabolism.

In summary, the downregulation of Glo1 activity via a Glo1 knockdown did not have any measurable effects on the weight, body composition, or metabolism. Furthermore, no significant effect on the formation of atherosclerotic lesions in the aorta in double transgenic Glo1^KD^ × ApoE^−/−^ mice could be detected.

## Supplementary Material

Supplementary Table 1: The names and sequences of the primers used for the genotyping of the transgenic mice used in this study are listed.
Supplementary Figure 1: Oxygen consumption (A), food intake (B) and activity (C) was analyzed during a 12h dark and a 12h light cycle. There was not significant effect of the Glo1 activity (p=0.05) on all parameters measured.
Supplementary Figure 2: Atherosclerosis of the whole aorta was measured via en face preparation. Bars indicate the plaque area in relation to the whole area in percent ± standard deviation. The Glo1 knockdown did not have a significant impact on the formation of atherosclerotic plaques (p=0.05).

## Figures and Tables

**Table 1:** The body composition was measured via Echo-MRI. There was no significant difference in the total body weight nor in the relative fat or lean mass (*p* ≥ 0.05).

Genotype	Diet	Weight (g)	Fat (%)	Lean (%)	Rest (%)
Glo1^KD^	High fat diet	40.8 ± 3.32	45.1 ± 3.0	49.5 ± 2.8	5.4 ± 0.5
Wild-type	High fat diet	42.4 ± 1.96	43.2 ± 0.9	50.9 ± 0.9	5.9 ± 0.8
Glo1^KD^	Low fat diet	27.9 ± 1.57	24.3 ± 4.6	67.4 ± 2.9	8.3 ± 1.8
Wild-type	Low fat diet	28.7 ± 2.44	22.2 ± 3.7	69.6 ± 3.6	8.2 ± 1.3
