# Supplementary material for: A Glyoxalase-1 Knockdown Does Not Have Major Short Term Effects on Energy Expenditure and Atherosclerosis in Mice
Source: J Diabetes Res. 2015 Dec 15;2016:2981639. doi: 10.1155/2016/2981639 (PMC4693023; doi:10.1155/2016/2981639)
Supplement: Supplementary file 1 — Supplementary Table 1: The names and sequences of the primers used for the genotyping of the transgenic mice used in this study are listed. Supplementary Figure 1: Oxygen consumption (A), food intake (B) and activity (C) was analyzed during a 12h dark and a 12h light cycle. There was not significant effect of the Glo1 activity (p=0.05) on all parameters measured. Supplementary Figure 2: Atherosclerosis of the whole aorta was measured via en face preparation. Bars indicate the plaque area in relation to the whole area in percent ± standard deviation. The Glo1 knockdown did not have a significant impact on the formation of atherosclerotic plaques (p=0.05). [file 2981639.f1.pdf]

Supplementary Table 1

Primer name	Sequence
Apoe-ko-for	5’-GCCTAGCCGAGGGAGAGCCG-3’
Apoe-ko-rev1	5’-TGTGACTTGGGAGCTCTGCAGC-3’
Apoe-ko-rev2	5’-GCCGCCCCGACTGCATCT-3’
Glo1-gd-for	5’-GCTTCTCCCACAAGTCTGTG-3’,
Glo1-gd-rev	5’-GGTACAGTGCAGGGGAAAGA-3’

Supplemental Figure 1

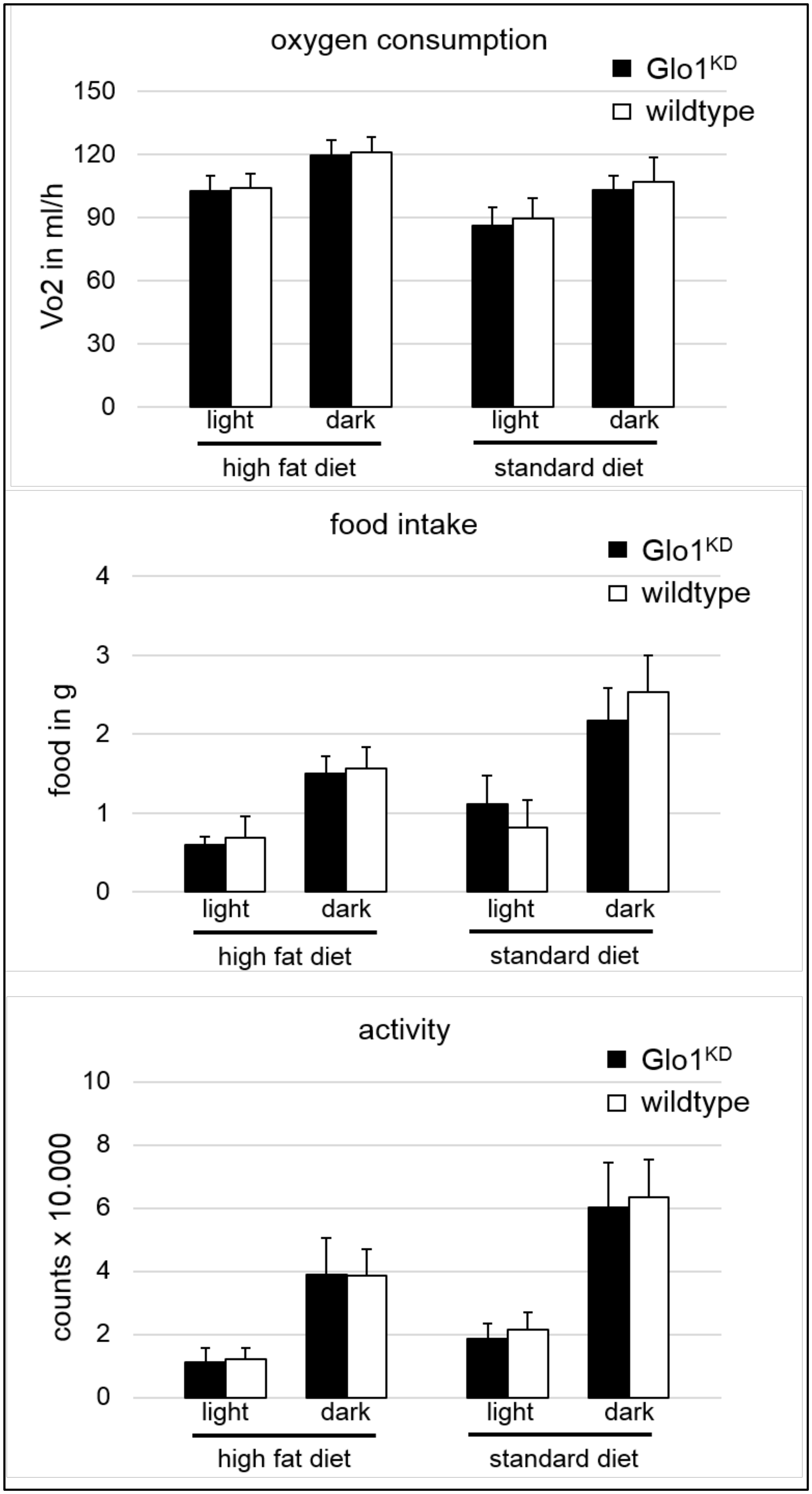

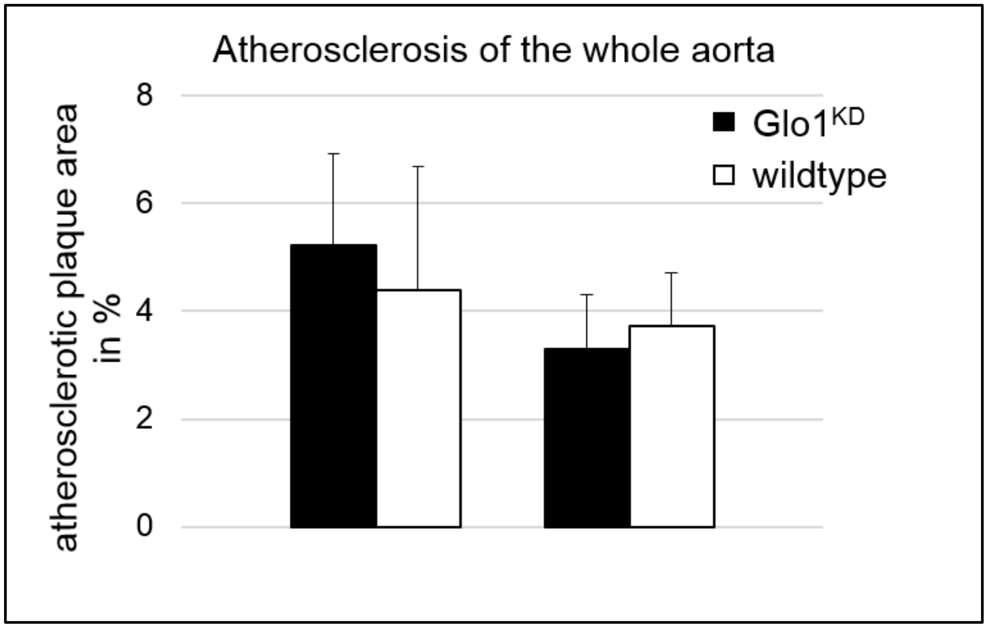

Supplemental Figure 2
